# 

**Published:** 1883-03

**Authors:** 


					MARCH, 1883.
THE
AMERICAN JOURNAL
op
I) K X T A L SCI E N C E,
EDITED BY
F. J. S. G0RGA8, M. D., D. D. S.
VOL. 16.?THIRD SERIES.?No ll.
n A L T J M O li K:
S X OW L) E N it C () W M A X.
LONDO X
TliUBNEfi & CO.. 60 l'ATEIi'NOST-ER ROW.
1 S ?> 3 .
payabuPn advanck.
PliBLMEU MONTHLY.
$2.50 PER ANNUM.

				

